# Multiple Novel Nesprin-1 and Nesprin-2 Variants Act as Versatile Tissue-Specific Intracellular Scaffolds

**DOI:** 10.1371/journal.pone.0040098

**Published:** 2012-07-02

**Authors:** Dipen Rajgor, Jason A. Mellad, Flavia Autore, Qiuping Zhang, Catherine M. Shanahan

**Affiliations:** 1 Cardiovascular Division, James Black Centre, King’s College London, London, United Kingdom; 2 The Randall Division of Cell and Molecular Biophysics, New Hunt’s House, King’s College London, London, United Kingdom; University of Missouri-Columbia, United States of America

## Abstract

**Background:**

Nesprins (Nuclear envelope spectrin-repeat proteins) are a novel family of giant spectrin-repeat containing proteins. The nesprin-1 and nesprin-2 genes consist of 146 and 116 exons which encode proteins of ∼1mDa and ∼800 kDa is size respectively when all the exons are utilised in translation. However emerging data suggests that the nesprins have multiple alternative start and termination sites throughout their genes allowing the generation of smaller isoforms.

**Results:**

In this study we set out to identify novel alternatively transcribed nesprin variants by screening the EST database and by using RACE analysis to identify cDNA ends. These two methods provided potential hits for alternative start and termination sites that were validated by PCR and DNA sequencing. We show that these alternative sites are not only expressed in a tissue specific manner but by combining different sites together it is possible to create a wide array of nesprin variants. By cloning and expressing small novel nesprin variants into human fibroblasts and U2OS cells we show localization to actin stress-fibres, focal adhesions, microtubules, the nucleolus, nuclear matrix and the nuclear envelope (NE). Furthermore we show that the sub-cellular localization of individual nesprin variants can vary depending on the cell type, suggesting any single nesprin variant may have different functions in different cell types.

**Conclusions:**

These studies suggest nesprins act as highly versatile tissue specific intracellular protein scaffolds and identify potential novel functions for nesprins beyond cytoplasmic-nuclear coupling. These alternate functions may also account for the diverse range of disease phenotypes observed when these genes are mutated.

## Introduction

Nuclear envelope (NE) spectrin-repeat proteins, or nesprins, are a novel family of nuclear and cytoskeletal proteins with rapidly expanding roles as intracellular scaffolds and linkers [1], [2], [3], [4]. Nesprins are characterized by a central extended spectrin-repeat (SR) rod domain and a C-terminal Klarsicht/ANC-1/Syne homology (KASH) transmembrane domain, which acts as a NE targeting motif. At the NE, via interactions with the Sun-domain family of proteins and the nuclear lamina, nesprins on both the inner and outer nuclear membrane form the linker of the nucleoskeleton and cytoskeleton (LINC) complex [5], [6]. This complex requires the giant nesprin-1 (∼1 MDa) and nesprin-2 (∼800 kDa) isoforms, which possess a pair of N-terminal calponin homology domains, which bind directly to F-actin [7], [8]. Nesprin-3 (∼110 kDa) and nesprin-4 (∼43 kDa) are smaller family members with more divergent spectrin-repeats. These lack the N-terminal CH domains of nesprin-1 and -2 and via SRs interact with intermediate filaments and microtubules respectively [3], [4], [9].

Disruption of the LINC complex via mutations in nesprin-1 and -2 or their binding partners, such as emerin and lamin A/C, give rise to Emery Dreifuss Muscular Dystrophy (EDMD) [6], [10], [11], [12], [13], [14], [15], [16], [17]. However, emerging evidence implicates nesprin-1 and -2 in several other unrelated diseases, including schizophrenia, epithelial cancers and autosomal recessive cerebellar ataxia (ARCA1), which are not characterized by NE defects [18], [19], [20]. It is likely that these non-canonical roles for nesprin are mediated by alternative transcription that has been shown to generate multiple tissue-specific nesprin variants that lack either the CH domain, the KASH domain or both and localize to a number of subcellular compartments [2], [21]. For example, nesprin-1 has been shown to localize to the Golgi apparatus and over-expression of dominant-negative nesprin-1 fragments composed of SRs within the central rod domain disrupt Golgi organization and function [22], [23], [24]. Nesprin-1 isoform Drop1, which consists of the N-terminal CH domain and SRs but lacks the KASH domain, is significantly down regulated in epithelial cancer and may play a role in chromatin organization [16], [25], [26]. Furthermore, the brain-specific nesprin-1 isoform, candidate plasticity gene 2 (cpg2), consists solely of SRs and localizes to the neuronal postsynaptic endocytic zone surrounding dendritic spines where it regulates clathrin-mediated uptake and recycling of chemokine receptors [27], [28].

In order to assess further the extent of alternate nesprin functionality, in this study we set out to identify novel nesprin variants by identifying 5′UTRs and 3′UTRs transcribed from the nesprin-1 and nesprin-2 genes. We provide evidence that both nesprin-1 and -2 undergo alternative splicing and express multiple tissue specific variants generated by alternate initiation and termination and that the sub-cellular localization of these variants is cell type dependent. We also provide a unifying nomenclature system for nesprin variants and their UTRs.

## Results

### Identification of Novel Nesprin-1 and Nesprin-2 UTRs

**Figure 1:**
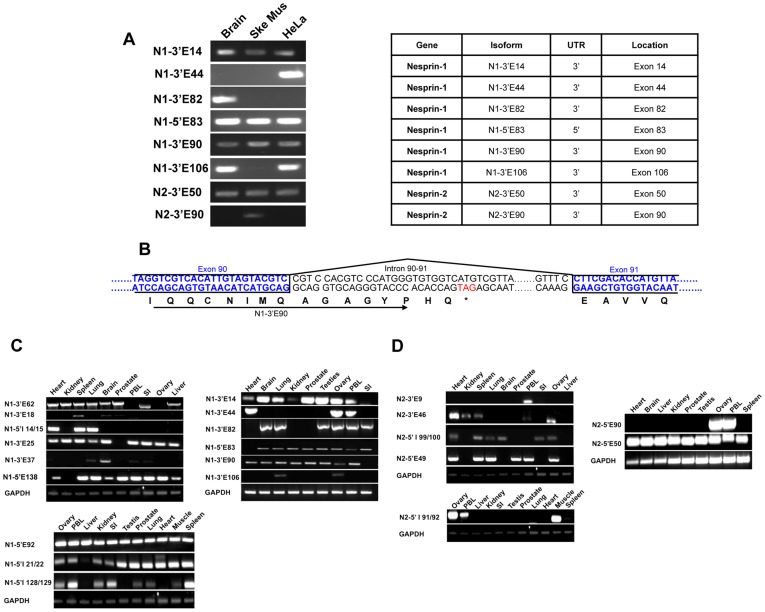
Identification of novel nesprin UTRs. A) cDNA ends identified by 3′ and 5′ RACE from Brain, Skeletal Muscle (SkeMus) and HeLa cDNA libraries. B) DNA sequencing results suggest that nesprin isoforms terminate with unique C-terminal ends absent from the giant isoforms as a result of intron retention. For example, isoforms utilising the N1-3′E90 UTR terminate with ‘AGAGYPHQ’ amino acids, giving it a unique fingerprint. Blue sequences show the coding regions of exons 90 and 91, black sequences show intronic regions and red sequence indicates a stop codon. C) Validation and tissue specificity of nesprin-1 UTRs identified on online databases and by RACE were confirmed by PCR amplification from a multiple tissue cDNA panel and DNA sequencing. Nesprin-1 PCRs were carried out when UTRs were identified on cDNA panels available at the time and are therefore organised into 3 separate sections. D) Validation and tissue specificity of nesprin-2 UTRs identified on online databases and by RACE were confirmed by PCR amplification from a multiple tissue cDNA panel and DNA sequencing. Nesprin-2 PCRs were carried out when UTRs were identified on cDNA panels available at the time and are therefore organised into 3 separate sections. Small Intestine and Peripheral Blood Lymphocytes have been abbreviated as ‘SI’ and ‘PBL’ respectively for all cDNA panels.

We adopted two approaches to identify *bone fide* novel 5′ and 3′UTRs. We first performed 5′ and 3′ RACE from HeLa, Skeletal Muscle and Brain cDNA libraries using multiple gene specific nesprin primers and nested primers designed towards the end of a range of exons throughout the nesprin gene. Many PCRs produced non-specific or no amplicons (data not shown) however, products for multiple UTRs as retained introns were detected (Figure 1A). For nesprin-1 we identified N1-5′E83, a novel 5′UTR where the first coding exon utilised is exon 83. Multiple nesprin-1 3′UTRs were detected in a tissue/cell specific manner where the last coding exons were either exons 14, 44, 82, 90 or 106 (N1-3′E14, N1-3′E44, N1-3′E82, N1-3′E90 and N1-3′E106 respectively). Similarly N2-3′E50 and N2-3′E90 are two novel nesprin-2 3′UTRs also identified by RACE. The stop codons for isoforms terminating with these novel 3′ ends were found in retained intronic sequences between the last coding exon and the exon thereafter. In nesprin variants using these 3′ ends the ‘retained intron’ resulted in the addition of unique amino acids followed by a stop codon that were absent from the giant isoforms. Downstream of the stop codon will be a polyA signal followed by a polyadenylation site. For example, variants terminating with the N1-3′E90 UTR contain eight codons before the stop codon in the retained intron between exon 90 and exon 91. Thus variants terminating with this 3′UTR possess a novel 8 amino acid peptide sequence, ‘AGAGYPHQ*’, which is absent in the giant isoform (Figure 1B).

Due to limitations with RACE analysis we next screened available databases for novel nesprin cDNA transcripts. The NCBI expressed sequence tag database (EST), which consists of one-shot sequences of cloned mRNA, was blasted with consecutive, 500 bp-overlapping 1 kb nesprin-1 and nesprin-2 sequences covering the entirety of the giant isoform cDNAs. Several novel UTRs were detected in the EST database screen, typically presenting as retained introns between two exons (Table 1). 5′UTRs were considered real if they contained an identifiable and viable Kozak sequence surrounding the first start codon. Only those 3′UTR sequences which already included a poly(A) tail or contained at least one poly(A) site downstream of the initial ORF termination codon, as determined by scanning with the polyAdq program or manually for non-canonical poly(A) signals, were considered for further study.

**Table 1:** UTRs identified through online databases.

Gene	UTR	5′ or 3′	Location	NCBI Accession	Verified by PCR
Nesprin-1	N1-5′I14/15	5′	Intron 14–15/	DA151121, CR933676, AK055440, BG197747, DB324328	+
Nesprin-1	N1-3′E18	3′	Exon 18	BC028616, DB545136, DB540697, DB538738	+
Nesprin-1	N1-5′I18/19	5′	Intron 18–19	DA337073	−
Nesprin-1	N1-3′E20	3′	Exon 20	DB540697, DB545136, DB538738	−
Nesprin-1	N1-5′I21/22	5′	Intron 21–22	DA337073	+
Nesprin-1	N1-3′E25	3′	Exon 25	DA151121, CR933676, AK055440, BG197747, DB324328	+
Nesprin-1	N1-3′E37	3′	Exon 37	AL713682	+
Nesprin-1	N1-5′E44	5′	Exon 44	DB300122	−
Nesprin-1	N1-3′E62	3′	Exon 62	BC039121,CA425673,AW300380, BG203678, BG210617, DB516174,CA441052, BX093712,CA312508, DB319424, AA227537,AI866946	+
Nesprin-1	N1-5′E84	5′	Exon 84	BU461222	−
Nesprin-1	N1-3′E87	3′	Exon 87	AB033088	−
Nesprin-1	N1-5′E92	5′	Exon 92	CJ462692, DA229059, DA227411, DA212433, DA509325, DB059554,DA802484, EE366817, DA241105, DA338782, DB289567, DA116814,DA493491	+
Nesprin-1	N1-5′E97	5′	Exon 97	BF740426, BF726175	−
Nesprin-1	N1-5′I128/129	5′	Intron 128–129	AK304825	+
Nesprin-1	N1-5′I132/133	5′	Intron 132–133	DA632075	−
Nesprin-1	N1-5′E138	5′	Exon 138	DA827648	+
Nesprin-2	N2-3′E9	3′	Exon 9	BC042134, BC071873	+
Nesprin-2	N2-3′E46	3′	Exon 46	BX648234	+
Nesprin-2	N2-5′E49	5′	Exon 49	BC036941, BI860943,AA247756	+
Nesprin-2	N2-5′E63	5′	Exon 63	CV571029	−
Nesprin-2	N2-5′I 91/92	5′	Intron 91–92	DA226447	+
Nesprin-2	N2-5′I 99/100	5′	Intron 99–100	DB089560, BM805144, DB088145, DA810994, DA725349, DA101036,DB152052, DA196319, DA706514, DA186088, DA106538, DA093934,DA334037, DA333629, DB063748, DA222451, DA230417, DA097798,DA094004, DA522676	+

Table listing all potential UTRs identified through available online databases.

**Figure 2:**
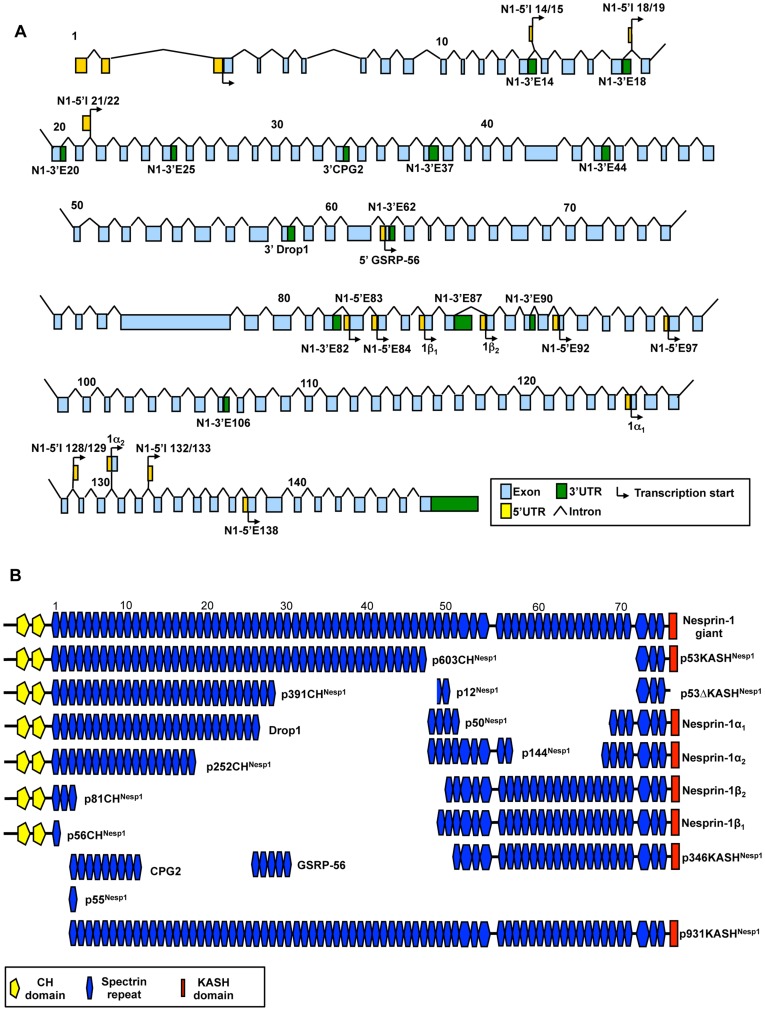
Potential nesprin-1 isoforms. A) Genomic map of the nesprin-1 gene highlighting the positions of the nesprin-1 UTRs identified to date. B) Proposed nesprin-1 isoforms created by alternative transcription. SRs are numbered and shown according to the scheme of *Simpson and Roberts 2008* and are shown to scale.

**Figure 3:**
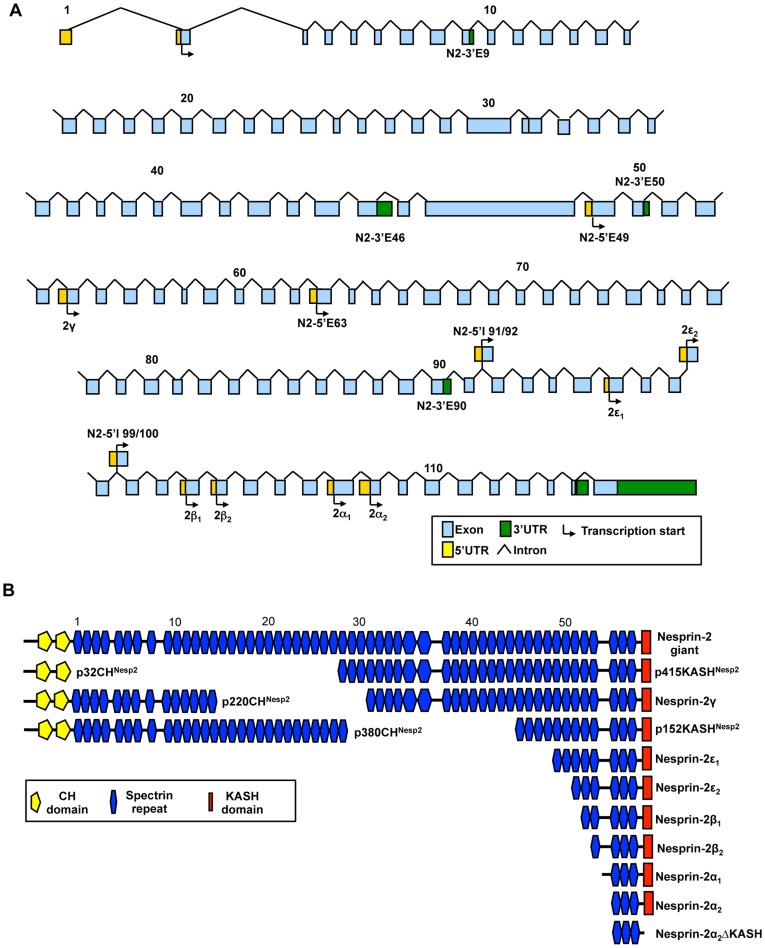
Potential nesprin-2 isoforms. A) Genomic map of the nesprin-2 gene highlighting the positions of the nesprin-1 UTRs identified to date. B) Proposed nesprin-2 isoforms created by alternative transcription. SRs are numbered and shown according to the scheme of *Simpson and Roberts 2008* and are shown to scale.

The majority of UTRs identified by RACE or through the EST screen were verified by PCR and DNA sequencing using a multi-tissue cDNA panel (Figures 1C,D. Table 1 contains a column showing the UTRs which have been verified by PCR). PCR primers were designed so that one primer was present within the UTR and the second within a constitutively present exon with at least one intervening intron sequence to control for genomic DNA contamination. Although many UTRs PCR amplified in a range of tissues, most were transcribed in a tissue specific manner suggesting they lead to the creation of tissue specific nesprin variants. The potential combinations of 5′ UTRs with 3′UTRs are extensive and would allow generation of many variants. Figures 2A and 3A provide an outline of the nesprin-1 and nesprin-2 UTRs across their respective genes with figures 2B and 3B highlighting proposed variants that could be created by a ‘mix-and-match’ approach *in vivo* for nesprin-1 and nesprin-2 respectively (Table S1 and Table S2 shows the UTRs that when combined generate these nesprin-1 and -2 variants respectively). The spectrin repeats (SRs) used in our schematics to represent nesprins are based on the predictions of SRs as previously described [29]. Many of the predicted nesprin variants are too large to be detected by conventional PCR and are therefore hypothetical. The smaller nesprin variants were however validated by PCR and are described below.

Although many variants could retain the KASH domain, there is a possibility of generating isoforms composed solely of SRs. Therefore, the identified nesprin variants were named according to their predicted molecular weights and the domains they possessed. For example, p56CH^Nesp1^ is a nesprin-1 variant of 56 kDa which has the N-terminal CH domains, p50^Nesp1^ is a 50 kDa nesprin-1 variant which lacks both the CH domains and the KASH domain and is composed of SRs, while p53KASH^Nesp1^ is a 53 kDa KASH containing variant lacking CH domains. Variants that lack the KASH domain due to alternative splicing events in and around the exons coding for the KASH domain have been described as ΔKASH variants (See below).

### Nesprin KASH Isoforms

**Figure 4:**
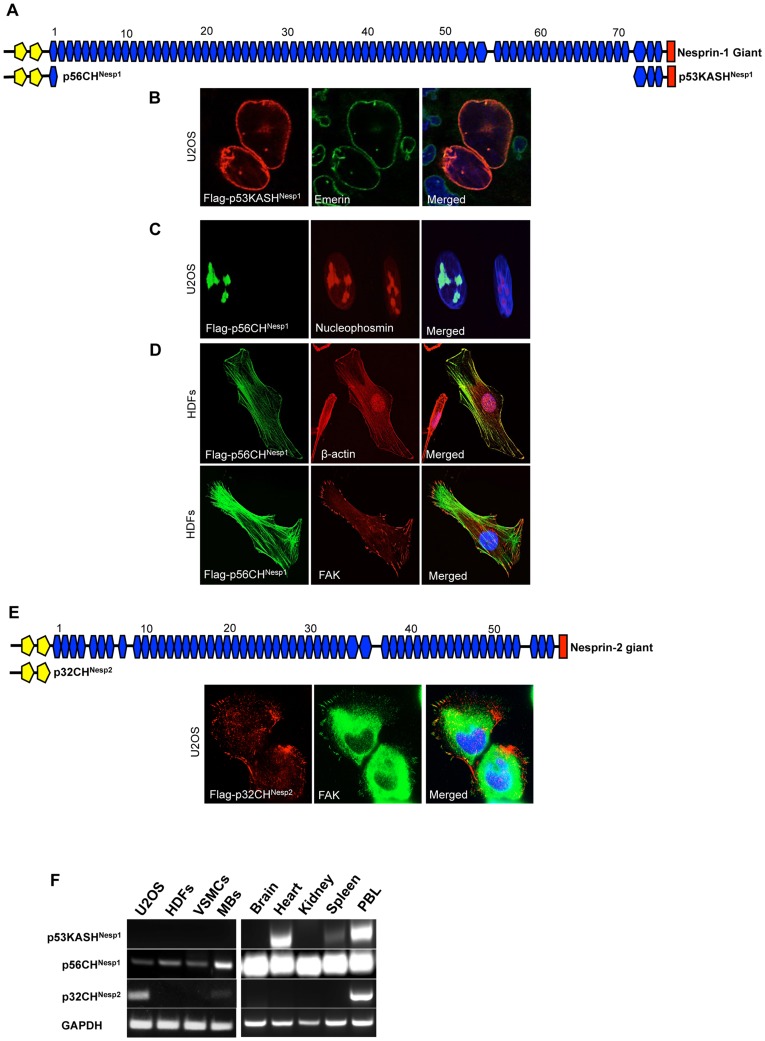
Cloning and expression of novel Nesprin KASH and CH isoforms. A) Schematic representation of p53KASH^Nesp1^ (Accession numberJQ754366) and p56CH^Nesp1^ (Accession number JQ740783) relative to the nesprin-1 giant. B) p53KASH^Nesp1^ localizes to the NE when transfected into U2OS cells. C) Nesprin-1 Flag-p56CH^Nesp1^ localized to the nucleolus when transfected into U2OS cells. D) Nesprin-1 Flag-p56CH^Nesp1^ localizes to actin stress fibres and with Focal Adhesion Kinase (FAK) at focal adhesions when transfected into Human Dermal Fibroblasts (HDFs). E) Nesprin-2 Flag-p32CH^Nesp2^ (Accession numberJQ754367) co-localized with FAK at focal adhesions when transfected into U2OS cells. F) p53KASH^Nesp1^ expression was not detected by PCR in U2OS, Human Dermal Fibroblasts (HDFs), Vascular Smooth Muscle Cells (VSMCs) or Myoblasts (MBs), however it was detected in the heart, spleen and peripheral blood leukocytes (PBL). p56CH^Nesp1^ was detected in all cells and tissues examined whereas p32CH^Nesp2^ was limited to U2OS cells, MBs and PBL.

So far a number of KASH variants including the nesprin-1 and nesprin-2 α,β isoforms have been identified. In principle any of the 5′UTRs identified in this study could be utilised with the 3′UTR of the nesprin-1 giant to make KASH containing NE localized nesprin variants. Whilst most 5′UTRs are too distant from the KASH domain for PCR amplification we were able to verify p53KASH^Nesp1^ (Accession number JQ754366), the smallest nesprin-1 KASH containing isoform identified to date, with a molecular weight of 53 kDa. p53KASH^Nesp1^ uses the N1-5′E138 alternative start site which was detected in heart, spleen, lung, brain, prostate, PBL, small intestine (SI), ovary and liver cDNA (Figure 1C). Although full-length p53KASH^Nesp1^ could not be detected in a range of primary and transformed cell lines it was detected in tissues including heart, spleen and peripheral blood leukocytes (PBL) (Figure 4F). Flag-p53KASH^Nesp1^ cloned from heart cDNA confirmed NE localization when transfected into U2OS cells (Figure 4A,B).

### Nesprin Calponin Homology Domain containing Isoforms

Next we set out to identify the sub-cellular localizations of KASH-less nesprin variants composed of SRs or CH domains. p56CH^Nesp1^ and p32CH^Nesp2^ are two nesprin CH-domain containing variants with a Mw suitable for PCR amplification and cloning (Accession numbers JQ740783 and JQ754367 respectively) (Figures 4A,E). p56CH^Nesp1^ initiates with the most upstream nesprin-1 UTR utilised by the nesprin-1 giant and terminates with N1-3′E14, encoding a protein that possesses the CH domains and the first SR of nesprin-1. p32CH^Nesp2^ terminates upstream of the first SR coding exon and is therefore the only known nesprin variant to date which lacks any SRs. Interestingly we observed differential sub-cellular localizations when p56CH^Nesp1^ was transfected into transformed and primary cell lines. In U2OS cells p56CH^Nesp1^ surprisingly localized to the nucleolus while in HDFs it associated with actin stress fibres and focal adhesions (Figure 4C,D). Whilst p56CH^Nesp1^ expression was ubiquitously detected in all cell lines examined, expression of p32CH^Nesp2^ was limited to PBL, MBs and U2OS cells (Figure 4F). Unlike p56CH^Nesp1^, p32CH^Nesp2^ localized to focal adhesions when ectopically expressed in its native U2OS cells (Figure 4E).

### Nesprin Central Rod Isoforms

**Figure 5:**
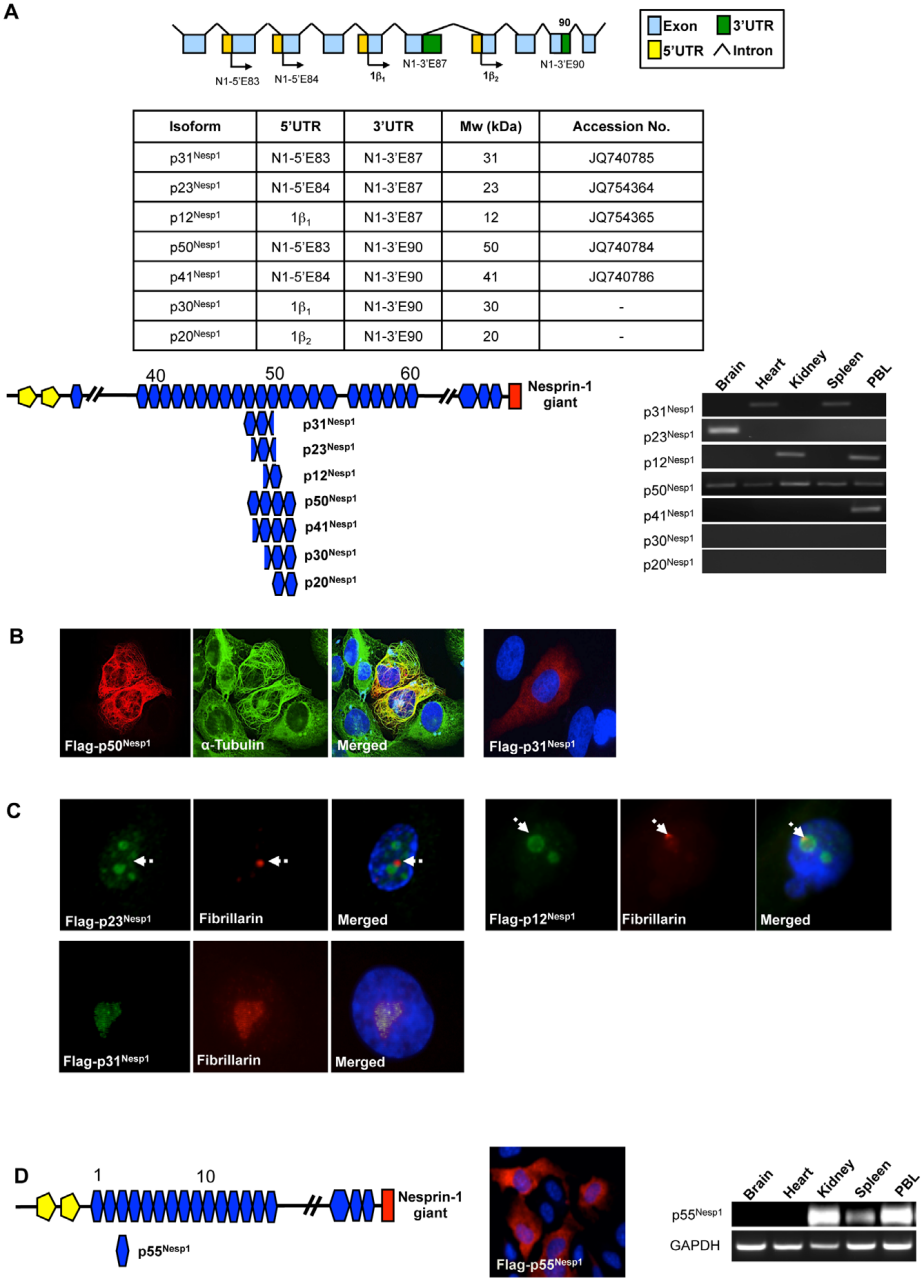
Nesprin-1 Central rod isoforms. A) Nesprin-1 isoforms p31^Nesp1^, p23^ Nesp1^, p12^ Nesp1^, p50^ Nesp1^, p41^ Nesp1^, p30^ Nesp1^ and p20^ Nesp1^ are potential variants which could be generated through alternative initiation and termination using UTRs located between exons 83 and 90. All isoforms except p30^Nesp1^ and p20^Nesp1^ PCR amplified from at least one tissue examined. B) p50^Nesp1^ localized to and polymerized microtubules in U2OS cells. p31^Nesp1^ displayed a diffusive localization when transfected into U2OS cells. See Figure S1A for diffusive localization staining of p23^Nesp1^, p12^Nesp1^ and p41^Nesp1^. C) p23^Nesp1^ and p12^Nesp1^ promoted nucleolar cap formation in HDFs while p31^Nesp1^ localized to the nucleolus without causing any nucleolar disruption. D) p55^Nesp1^ localized diffusively around the cytosol when transfected into U2OS cells and was detected in the kidney, spleen and peripheral blood leukocytes (PBL) by PCR.

Multiple 5′ and 3′ UTRs were identified in the nesprin-1 gene between exons 83 and 90, suggesting that it is a region where multiple variants are generated (Figure 5A). Using RACE we identified a 5′UTR where the first coding exon was exon 83 (N1-5′E83) and a 3‘UTR where the final coding exon was exon 90 (N1-3′E90) (Figure 1A). Furthermore online databases revealed an additional 5′UTR associated with exon 84 (N1-5′E84) and a previously described Kazusa clone KIAA1262. The KIAA1262 sequence includes exons 77 to 87 and terminates in a 3′UTR where the final coding exon is exon 87 (N1-3′E87). The identification of these new UTRs together with the pre-existing nesprin-1_β1_ and nesprin-1_β2_ 5′UTRs confirms that this is a region of nesprin with the ability to generate multiple alternative transcripts (Figure 5A).

Hypothetically these UTRs could generate 7 nesprin-1 splice variants by alternative initiation and termination of the four 5′UTRs with the three 3′UTRs (Figure 5A). To test this, PCR amplification from 5′ to 3′UTRs were carried out in multiple tissue cDNA panels to see if any of the variant messages were transcribed. p50^Nesp1^ (Accession number JQ740784) expression was ubiquitous while expression of the p41^Nesp1^ (Accession number JQ740786), p31^Nesp1^(Accession number JQ740785), p23^Nesp1^ (Accession number JQ754364) and p12^Nesp1^ (Accession number JQ754365) variants was restricted to certain tissues. p30^Nesp1^ and p20^Nesp1^ failed to amplify and therefore are probably not expressed (Figure 5A). When p50^Nesp1^ was expressed in U2OS cells it localized to and polymerized microtubules while all the other isoforms displayed a diffuse cytoplasmic and nuclear localization (Figure 5B for Flag- p50^Nesp1^ and Flag- p31^Nesp1^). All other isoforms are shown in Figure S1A). p23^Nesp1^ and p12^Nesp1^ both localized to and disrupted nucleolar morphology when expressed in HDFs, causing fibrillarin to redistribute into peri-nucleolar caps, while the slightly larger p31^Nesp1^ localized with fibrillarin without affecting its localization (Figure 5C). When p41^Nesp1^ was expressed in HDFs, it displayed diffuse cytoplasmic localization and also concentrated around the ER (Figure S1B).

Another central rod variant Nesprin-1 p55^Nesp1^, is composed of a single SR and lacked both the CH and KASH domains (Figure 5D). p55^Nesp1^ was detected in the kidney, spleen and PBL by PCR and displayed diffuse cytoplasmic localization when transfected into U2OS cells (Figure 5D).

### Nesprin Isoform Expression is Highly Adaptable

**Figure 6:**
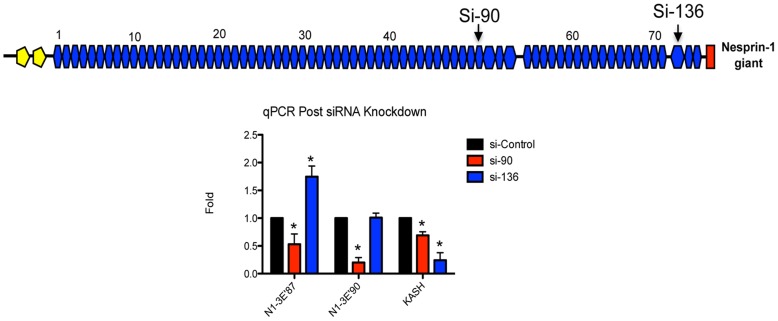
Nesprin-1 expression is highly adaptable. Expression levels of N1-3′E87, N1-3′E90 and nesprin-1 KASH domain were monitored post-siRNA knockdown using siRNAs targeting exons 90 and 136 of the nesprin-1 gene. As demonstrated si-136 increased expression of N1-3′E87 whereas si-90 reduced it’s expression. *p<0.01, ANOVA analysis, 95% confidence interval.

To further confirm the validity of the novel variants and because previous evidence indicates that nesprins have the ability to self-compensate we decided to investigate how knocking down a sub-set of transcripts would effect expression levels of variants encoded by nearby transcripts [30]. By designing an siRNA targeting exon 90 (si-90) we were able to monitor by qRT-PCR the levels of transcripts terminating with N1-3′E87 and N1-3′E90 UTRs (Figure 6). In theory this siRNA should target all transcripts terminating with N1-3′E90 but have no effect on N1-3′E87 terminating transcripts as this termination site is located to the 5′ end on exon 87. As expected, si-90 significantly reduced levels of N1-3′E90 expression but more interestingly also significantly knocked down levels of the transcripts terminating with N1-3′E87. Furthermore si-136, an siRNA designed towards the KASH domain of nesprin-1 increased expression of N1-3′E87 transcripts, showing that perturbations in the expression of one transcript can influence expression of other downstream transcripts. Conversely no change in N1-3′E90 was detected with si-136, however both siRNAs knocked down levels of nesprin-1 KASH expressing transcripts (Figure 6).

### Nesprin and Alternative Splicing of Cassette Exons

Next we set out to determine whether any of the 100 plus exons of the nesprin-1 and nesprin-2 genes have the ability to undergo alternative splicing to increase further variant diversity. As a starting point we used nesprin EST and nucleotide databases to look for potential splicing events which identified cassette exons 6′, 93 and 145 for nesprin-1 (Table 2). Exon 6′ is a potential isoform or tissue specific coding region located between exons 6 and 7 and encodes a 7 amino acid peptide insert at the end of the first CH domain of nesprin-1 (See Figure S2A for nesprin-1 genomic map with cassette exon). Exon 93 contributes an additional 47 amino acids to a SR of the central rod domain while exon 145 encodes a peptide sequence at the C-terminal region of the KASH domain.

**Table 2:** Cassette exons identified through online databases.

Nesprin	Exon	Splicing	Peptide Sequence	Accession
Nesprin-1	Exon 6′	Cassette	SMHRGSP	CF552114
Nesprin-1	Exon 93	Cassette	MTAGRCHTLLSPVTEESGEEGTNSEISSPPACRSPSPVANTDASVNQ	DB289567,AK310977,CA425673,CA312508,DA809350,DB224830,DA493464
Nesprin-1	Exon 145	Cassette	VHKRWLRFLPF*	BX647837
Nesprin-2	Exons 28–31	Cassette	Premature stop codons	AU185086
Nesprin-2	Exon 101’	Cassette	PTHGVQQKYYLMMTKNAMFIREEVFQFFPMTMHFLFINVIFPKLGNCITIIIKGQDSRDPTSLQATTALAGLYQLGRQGSTARY	CR749324
Nesprin-2	Exon 107’	Cassette	DVEIPENPEAYLKMTTKTLKASS	NM_182914,DA044815,DB138084,DA868743
Nesprin-2	Exons 110–113	Cassette	IRASSPSKVQSSENYRRRGGDREQGPRQHTATALLPLKGGPGSPTPAAAPPAAAAPGLPAALLRRRLQLHSGQQLCPVLLPHAEVHQWATPHIEGIAGHSATPPA*	BM725084
Nesprin-2	Exon 114	Cassette	NPASPLPSFDEVDSGDQPPATSVPAPRAK	BE795270

**Figure 7:**
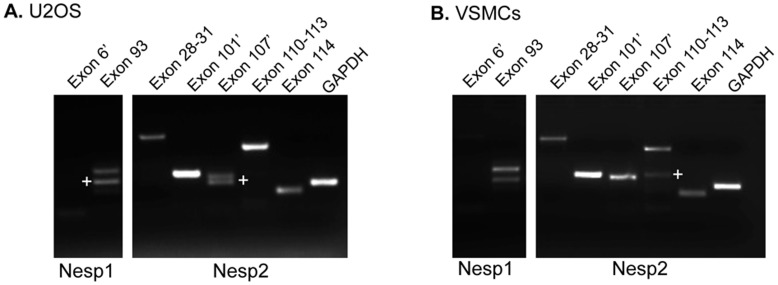
Identification of nesprin-1 and nesprin-2 splicing events. A) PCR amplification across splice sites was carried out from cDNA isolated from U2OS cells. Splicing of exon 93 for nesprin-1 was observed as was the splicing for nesprin-2 exon 107’. B) PCR amplification across splice sites was carried out from cDNA isolated from VSMCs. Splicing of exon 93 for nesprin-1 was observed. Exon 107’ was retained in all nesprin-2 transcripts while splicing of exons 110–113 was also observed in these cells. ^+^Represents bands with exon(s) excluded.

An online scan of the EST and nucleotide databases indicated that the nesprin-1 and nesprin-2 genes underwent extensive alternative splicing and this was verified using PCR (Figure 7A,B).

* Represents a stop codon for nesprin-1 exon 145 and nesprin-2 exons 110–113.

Several alternatively spliced exons, were also identified in nesprin-2 using the same data mining procedure, including cassette exons 101′, 107′ and 114 and 5′ alternatively spliced exons 110 to 113 (Table 2). Unlike nesprin-1, the identified exons were all located near the C-terminal half of the nesprin-2 giant. Splicing of exons 101′, 107′, 110 to 113 and 114 would alter the length of the coiled-coil regions surrounding the two SR preceding the KASH domain while removal of the first 31aa encoded by exon 113 would eliminate the final SR before the KASH domain. As with exon 6′ of nesprin-1, exon 101′ and 107′ of nesprin-2 represent coding regions which maybe isoform or tissue specific and are located between exons 101 to 102 and 107 to 108 respectively. (See Figure S2B for nesprin-2 genomic map with cassette exons).

To identify whether these splicing events did indeed take place we designed primers to exons either side of the cassette exons and carried out PCR analysis from U2OS and VSMC cDNA libraries (Figure 7A,B). For nesprin-1, two PCR products appeared from U2OS and VSMC libraries when PCR amplification was carried out across exon 93; the larger of the two bands included exon 93 and the smaller band with the exon excluded. Nesprin-2 splicing showed more tissue specificity than nesprin-1, with PCR products including and excluding cassette exon 107′ expressed in U2OS cells at equal quantities while in VSMCs exon 107′ was exclusively expressed with no band detected for transcripts with the exon excluded expressed. Furthermore in VSMCs, transcripts with exons 110–113 removed were detected as well as transcripts with the exons included, although transcripts with the exons included seem to be transcribed in greater abundance. U2OS cells only expressed transcripts with the exons included.

Although splicing of cassette exons 6′ of nesprin-1 and exon 28–31, 101′ and 114 for nesprin-2 failed to be detected in this set of PCRs, examination of a wider array of cells and tissues is required to determine whether the splicing events listed in the databases occur.

### Nesprin ΔKASH Isoforms

**Figure 8:**
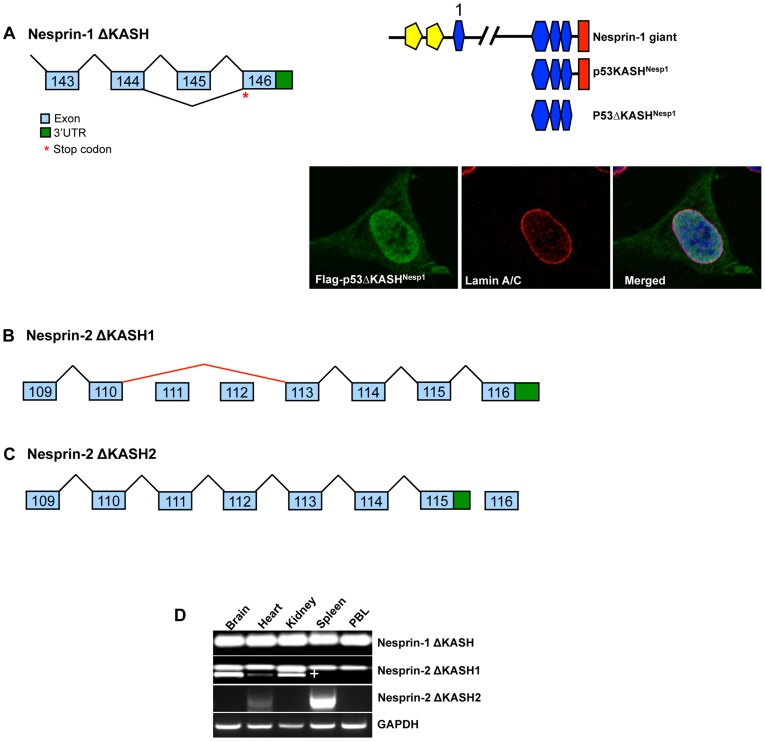
Generation of nesprin-1 and nesprin-2 ΔKASH variants. A) Nesprin-1ΔKASH is generated through the removal of cassette exon 145, resulting in disruption of the KASH domain. Ectopically expressed p53ΔKASH^Nesp1^ fails to localize to the NE in U2OS cells and is strongly concentrated within the nucleus and weakly in the cytosol. B) Nesprin-2ΔKASH1 is generated though the removal of exons 111–112 through the splicing event described in the previous section (splicing shown in red). C) Nesprin-2ΔKASH2 is generated through utilization of an alternative 3′UTR juxtaposed to exon 115. D) PCR-based tissue screen for ΔKASH variants shows that the removal of exon 145 for Nesprin-1ΔKASH is detected in a wide array of tissues. Nesprin-2ΔKASH1 splicing is detected pre-dominantly in the brain and kidney with small amounts also detected in the heart. ^+^denotes the spliced Nesprin-2ΔKASH1 product. Nesprin-2ΔKASH2 was detected in the heart and spleen only. Peripheral Blood Leukocytes (PBL).

The search for potential splicing events for nesprin also revealed splice events that eliminate the KASH domain. Alternative splicing of cassette exon 145 of nesprin-1, results in a frame shift that removes the KASH domain to create Nesprin-1ΔKASH (N1-ΔKASH) (Figure 8A). Though the same 3′UTR adjacent to exon 146 is shared between KASH domain and N1-ΔKASH sequences, the removal of exon 145 results in a change in the open reading frame of N1-ΔKASH variants and therefore they terminate with a novel 11aa tail: VHKRWLRFLPF rather than the RYTNGPPPL sequence utilized by KASH containing variants. Expression of N1-ΔKASH isoforms was detected in all tissue cDNA examined by PCR, suggesting that this splicing and resultant variants are ubiquitously expressed (Figure 8D). When the ΔKASH variant of p53KASH^Nesp1^ (p53ΔKASH^Nesp1^) lacking exon 145 was transfected into U2OS cells it no longer resided at the NE, but instead displayed strong nuclear matrix localization and weak cytoplasmic staining (Figure 8A).

Unlike N1-ΔKASH, nesprin-2 possesses two mechanisms for creating ΔKASH variants (Figure 8B,C). Like nesprin-1, generation of N2-ΔKASH1 occurs via the removal of cassette exons 110 to 113 but uses the same 3′UTR as the KASH variant. This splicing event results in a change in the ORF and therefore N2-ΔKASH1 terminates with a GIAGHSATPPA amino acid sequence rather than the YPMLRYTNGPPPT sequence used by KASH containing isoforms. N2-ΔKASH2 is created by a novel 3′UTR immediately adjacent to the 3′ end of exon 115. N2-ΔKASH2 splicing truncates larger isoforms without generating a novel C-terminal peptide. The N2-ΔKASH1 splicing was pre-dominantly detected in the brain and kidney with smaller amounts being amplified from the heart, where the lower band represents the removal of the cassette exons promoting N2-ΔKASH1 formation (Figure 8D). The N2-ΔKASH2 termination was detected in the heart and spleen only (Figure 8D).

## Discussion

### Nesprins as Adaptable, Tissue Specific, Intracellular Scaffolds

This study has demonstrated that nesprins, by generating variants via alternative transcriptional initiation and termination show localizations and therefore functions independent of their original description as NE linkers and scaffolds. Although nesprin-1 has the potential to generate more isoforms than nesprin-2, with more UTRs spread across the gene, the nesprin-2 isoforms are primarily N-terminal truncations that would retain the KASH domain. This suggests that nesprin-1 may have more functions beyond the NE than nesprin-2 or that sequences near the highly conserved C-terminus of nesprin-2 are important for cell function [29]. To our knowledge, the potential combinations of UTRs and exon splicing are unlimited. This combined with the ability of nesprins to dynamically regulate variant expression would allow cells to fine-tune their nesprin isoform repertoire as needed to maintain and restore homeostasis following stress or to regulate tissue-specific signalling pathways [31]. As a proof of principle we were able to show that nesprin transcription appears to be highly adaptable with a feedback loop regulating nesprin variant expression. For example we demonstrate using siRNAs that by knocking down a region of nesprin-1 near the KASH domain we were able to upregulate expression of N1-3′E87 UTR transcripts. Furthermore nesprin-2 CH domain knockout mice display an altered expression pattern for specific nesprin-2 C-terminal isoforms in certain tissues to compensate for the loss of nesprin-2 giant or nesprin-2 actin binding domain isoforms [30]. These observations suggest that nesprin alternative transcript generation is highly flexible and more complex than a simplified tissue-specific expression model.

### Generation, Regulation and Function of Novel Tissue Specific Nesprin Variants via Alternative Initiation and Termination

Using 5′ and 3′RACE as well as sequences in the EST database, followed by PCR amplification and DNA sequencing, we identified multiple novel sequence variants for nesprin-1 and -2. RT-PCR was used to establish the existence of mRNA transcripts for full-length short isoforms or for the novel UTRs of potentially larger variants. The multiple UTRs allow nesprins to express a large number of sequence variants via alternate initiation and termination and many of these were generated in a tissue specific manner. Therefore, in addition to the novel UTR’s verified in this study it is likely that by performing RACE in a greater collection of cells/tissues we may be able to identify further nesprin alternate initiation and termination sites.

To date, the mechanisms of tissue specific generation of nesprin variants has not been studied. Analysis of the human transcriptome shows a direct correlation between alternative promoter use and alternative splicing [32]. Alternative promoters can produce mRNAs with different 5′UTRs that encode the same protein, distinct N-termini, and even different proteins from the same locus [33], [34], [35], [36]. The identification of multiple, novel 5′UTRs in both nesprin-1 and -2 indicated the presence of several internal alternative promoters. The existence and regulation of alternative nesprin promoters has not yet been explored but this study suggests that these promoters are utilised in a tissue specific manner. Furthermore, because many individual variants have unique pairs of 5′ and 3′UTRs, additional control and regulation of variant expression can be maintained. The 5′UTR is an important regulator of mRNA translation and can contain regulatory motifs/sequences which affect the rate of translation as well as containing a kozak sequence upstream of the start codon which plays a major role in determining the translational strength of the transcript [37], [38]. The 3′UTR of mRNA transcripts can play a role in mRNA localization, stability, and translation [39], [40], [41], [42]. For example, binding of miRNAs to partially complementary sequences in the 3′UTR can result in de-adenylation and translational inhibition or destruction of the target mRNA [43], [44]. A RegRNA scan of both the nesprin 5′ and 3′UTRs for regulatory RNA motifs detected several potential miRNA binding sites which were transcript specific and could potentially regulate variant translation (data not shown).

Importantly, we showed that many of the variants generated through retained introns had generated unique peptide sequences. It is highly likely that these sequences expose new functional domains that give the variants additional localization signals or motifs that play an important role in their function. For example, ELM analysis predicts a novel retinoblastoma (Rb) interacting motif found in cell cycle regulatory proteins at the C-terminal end of p220CH^Nesp2^ while the N-terminus of p931KASH^Nesp1^ contains a potential N-myristoylation site, a post-translational modification which facilitates membrane anchoring [45], [46], [47]. ELM analysis also predicts multiple PKA, MAPK and CDK phosphorylation sites in the unique sequences of p32CH^Nesp2^, p56CH^Nesp1^ and p55^Nesp1^. To further explore this hypothesis yeast-2-hybrid analyses or co-immunoprecipitation assays will be required to identify binding partners for specific nesprin variants.

The ultimate validation for each proposed variant will be the detection of their translation and expression by western blotting. Post-translational modifications such as phosphorylation, sumoylation, and enzymatic cleavage may be partially responsible for the range of western bands often visualized using the currently available anti-nesprin antibodies [1], [17], [48]. Designing isoform-specific antibodies will help to distinguish between modifications and splicing.

### Alternative Splicing of Cassette Exons may Diversify Nesprin Function and Localization

In addition to alternative initiation and termination, we showed that some of the cassette exons located in the EST database are indeed valid. It is unclear whether these splice events occur in multiple variants or are isoform specific but they are likely to substantially increase nesprin diversity, likely in a tissue specific manner. This notion was supported by the observation that while some splice events occurred in the majority of cell lines, some events seemed to be cell type specific. For example unlike the nesprin-1 ΔKASH which was detectable in all cells tested, the splicing event of cassette exons which generate nesprin-2 ΔKASH seem to be very tissue specific. Furthermore the ability for nesprin-2 to generate ΔKASH variants via 2 different mechanisms, one by the utilization of a unique 3′UTR and another by the splicing of exons 110–113 suggest that the C-terminal ends of the ΔKASH variants may serve unique tissue specific functions at sites away from the NE.

Although in this study we did not look specifically at the effects of structure, function and localization of nesprin variants with and without alternate splicing of the cassette exons we did show that some of these splicing events also created unique peptides. Nesprin-1 exon 93 encodes a unique 47 amino acid peptide sequence and nesprin-2 exon 107′ encodes a unique 23 amino acid peptide. Although these peptide sequences are not very large they may be capable of encoding novel localization signals or binding sites for interactions with other proteins. Our next aim will be to create nesprin variants with and without these exons so we can identify their putative roles in nesprin function.

However we were able to show that KASH-less nesprin isoforms displayed subcellular localizations which varied depending on the cell lines they were expressed in. In U2OS cells p56CH^Nesp1^ localized to the nucleolus while the same protein localized along actin cables and focal adhesions in HDFs. Currently we do not understand what determines this differential change in subcellular localizations but we speculate that the presence of endogenous p32CH^Nesp2^ at focal adhesions in U2OS cells (p32CH^Nesp2^ is not expressed in HDFs but is expressed in U2OS) is enough for p56CH^Nesp1^ function to become redundant at focal adhesions in U2OS. Furthermore we suspect that differences in the actin cytoskeleton may play a role in differential localization of p56CH^Nesp1^. The nesprin-1 CH domains contain two nuclear localization signals which may be utilized in cells with low actin levels such as U2OS cells but not in structural cells such as HDFs where there is plenty of actin for the protein to bind to [49]. Alternatively, potential p56CH^Nesp1^ phosphorylation events predicted by ELM analysis may occur in a tissue specific manner which may contribute to the differences seen in localizations between the two cell types. Similarly differential sub-cellular localizations were seen when central rod isoforms p12^Nesp1^, p23^Nesp1^ and p31^Nesp1^, were transfected into U2OS and HDFs. In U2OS cells all isoforms displayed diffuse cytoplasmic localization while in HDFs nucleolar localization was observed. Differences in post-translational modifications could vary between the two cell lines or the proteins may have different binding partners in each cell line which could contribute to differential localizations. Ultimately the localization of these isoforms would have to be monitored in cells that express the variant endogenously.

### Isoforms and Disease

Multiple nesprin mutations have been identified in Emery Dreifuss Muscular Dystrophy (EDMD), Dilated Cardiomyopathies (DCM), autosomal recessive arthrogryposis (ARA) and autosomal recessive cerebellar ataxia (ARCA1) [15], [17], [18], [50], [51]. The nesprin mutations promoting EDMD and DCM presumably affect LINC nesprin variants resulting in abnormal nuclear morphology and function.

ARA is caused by an A to G mutation in a splice acceptor site, resulting in retention of intron 136 of nesprin-1 [51]. This mutation produces a premature stop codon and therefore the nesprin-1 giant, nesprin-1β and nesprin-1α isoforms should lack the KASH domain. Conversely these patients appeared to have no defects in nuclear morphology or lamin and emerin localization, suggesting other nesprin gene products or p53KASH^Nesp1^ which should not be effected by this mutation maybe enough to keep the nucleus intact.

ARCA1 is a neurological disease characterized by irregular gait and lack of limb coordination [18]. Five different mutations giving rise to ARCA1 were identified within the central spectrin rod of nesprin-1, upstream of the KASH variants identified so far. The A310067G mutation which effects the invariant A of the AG splice acceptor site at the junction of exon 85 and intron 84 results in the formation of a pre-mature stop codon and therefore will effect production of not only the p40, p50, p31 and p23 nesprin-1 proteins identified in this study but also other variants terminating with the N1-3′E87 and N1-3′E90 ends in there native tissues/cells. Although many diseases associated with nesprins so far suggest that NE nesprins are involved, ARCA1 patients appear to have no nuclear defects, suggesting that nesprin associated signalling pathways beyond the NE may be significantly hindered and potentially causative in the disease.

## Materials and Methods

### Identification of Novel UTRs

Rapid Amplification of cDNA Ends (RACE) on Brain, HeLa and Skeletal muscle Marathon-Ready cDNA libraries using the Advantage-2 PCR kit (Clontech) and gene specific primers was performed (Table S3). Resultant PCR fragments were cloned into pGEM-T easy vector (Promega) and sequenced (Gene Service). These sequences were then BLASTED against the human genome and novel cDNA ends were further analyzed.

Additional novel UTRs for nesprin-1 and nesprin-2 were identified by screening the NCBI expressed sequence tag (EST) database with consecutive, 500 bp-overlapping 1 kb Nesprin-1 and Nesprin-2 sequences covering the entirety of the giant isoform cDNAs. Tissue specificity of novel UTRs was determined by performing PCR amplification in a multiple tissue cDNA collection (Clontech). 30 PCR cycles were performed on 0.5 ul of cDNA followed by a further 15 cycles on 1 ul of the amplified product. Specificity of the PCR products were validated by DNA sequencing. Primers used for UTR expression can be found in Table S4. Primers used for cassette exon splicing and detection of nesprin ΔKASH variants can be found in Tables S5 and S6 respectively.

### Plasmids

Isoforms p53KASH^Nesp1^, p56CH^Nesp1^ and p50^Nesp1^ were *Taq* PCR amplified from tissue cDNA and cloned into pGEM-T Easy. The pGEM-T plasmids were subsequently used as templates for *Pfu* amplification with primers containing restriction sites and ligated into pCMV-Tag2 vector. p53ΔKASH^Nesp1^ was cloned using inverse PCR with *Pfu* off the Flag-p53KASH^Nesp1^ vector while p50^Nesp1^ served as a template for the inverse PCR and creation of p41^Nesp1^ and p30^Nesp1^. The Kazusa cDNA clone KIAA1262 served as a template for PCR amplification and cloning of p31^Nesp1^, p23^Nesp1^ and p12^Nesp1^ into a pCMV-Tag2 (Clontech). p32CH^Nesp2^ was PCR amplified from *IMAGE clone* 5478637 and cloned into pCMV-Tag2 as described.

### Tissue culture

Normal human dermal fibroblasts (HDF) and osteosarcoma cells (U2OS) were obtained from American Tissue Culture Collection (ATCC). The cells were passaged after reaching 70% confluency and maintained in DMEM complete media (Sigma) supplemented with 10 units/mL penicillin, 10 mg/mL streptomycin, 200 µM L-glutamine and 10% FBS.

### Transfections

For Flag-tagged construct expression, 1×10^6^ HDFs or U2OS cells were electroporated with 1 µg plasmid DNA using an Amaxa Nucleofector and cultured on coverslips for 16 hours.

### Immunofluorescence

Cells were fixed for 5 minutes in 3.7% paraformaldehyde in PBS followed by 2 min permeabilization with 0.5% NP-40 in PBS. The coverslips were incubated with blocking solution (1% BSA) for 1 hour at RT. The primary antibodies were diluted in blocking solution and applied to the coverslips for 1 hour at RT, followed by a 1 hour RT incubation with fluorescent dye-conjugated secondary antibodies (Invitrogen) diluted in blocking solution. The coverslips were washed with PBS, mounted onto slides with medium containing DAPI, and visualized using a Leica SP5 confocal microscope or a Zeiss Axioskop microscope.

### siRNA Knockdown and qRT-PCR

U2OS cells were transfected with siRNAs for nesprin-1 or control siRNA using hiperfect transfection reagent (Quiagen) as described by the manufacturer. Three days post-transfection total RNA was obtained from cells using Triazole RNA STAT-60 and phenol chloroform extraction. 2 µg of total RNA was reverse transcribed using AMV Reverse Transcriptase (Promega) according to the manufacturer’s instructions. qPCR was performed in a 20 µl reaction containing cDNA per 1× SYBR Green PCR master mix (Eurogentec) and 0.1 µM of each primer. PCR products were amplified, N1-3′E87 primers (forward, 5′- TCTCCAAGCTCAATCAGGCAGCAT -3′ and reverse, 5′- CACAGCCCTCTAAGTGTTGTGTCA -3′), N1-3′E90 primers (forward, 5′- AGTTGGACGTCTCAGTCTCAAGGA -3′ and reverse, 5′- TTTGATGGCTGAGCCCACACAATG -3′), KASH primers (forward, 5′-CGAGGCAAGTGTAGTCTCTCACAG-3′ and reverse, 5′-AGGGCCATTCGTGTATCTGAGCAT-3′) and GAPDH primers (forward, 5′-CGACCACTTTGTCAAGCTC-3′ and reverse, 5′- CAAGGGTCTACATGGCAAC-3′). The cycling parameters were 94°C for 15 seconds followed by a single step annealing and extension at 60°C for 60 seconds. Amplifications were performed on RotorGene-3000 (Corbett). Fold changes between samples were calculated by the delta-delta CT method.

## Supporting Information

Figure S1 **Localizations of p23^Nesp1^, p12^Nesp1^ and p41^Nesp1^.** A) p23^Nesp1^, p12^Nesp1^ and p41^Nesp1^ displayed diffusive cytoplasmic localization when transfected into U2OS cells. B) p41^Nesp1^ displayed diffusive localization and concentrated around the ER when transfected into HDFs.
(TIF)

Figure S2 **Schematics of nesprin-1 and nesprin-2 cassette exons.** A) Nesprin-1 genomic map showing the location of nesprin-1 cassette exon 6′ (Red box). B) Nesprin-2 genomic map showing the location of nesprin-2 cassette exons 101’ (orange box) and107’ (Purple box).
(TIF)

Table S1 **UTR combinations used to generate potential nesprin-1 variants.** Nesprin-1 can generate multiple variants through the use of alternative UTRs in a ‘mix-and-match’ approach. The tables highlight the UTR pairs used to generate the potential isoforms described in Figures 2B.
(DOCX)

Table S2 **UTR combinations used to generate potential nesprin-2 variants.** Nesprin-2 can generate multiple variants through the use of alternative UTRs in a ‘mix-and-match’ approach. The tables highlight the UTR pairs used to generate the potential isoforms described in Figures 3B.
(DOCX)

Table S3 **Primers used for 5′ and 3′ RACE.** Primers and nested primers used for detection of novel nesprin-1 and nesprin-2 cDNA ends.
(DOCX)

Table S4 **Primers used for UTR detection.** Forward and reverse primers used for detection of novel nesprin-1 and nesprin-2 UTRs. Forward and reverse primers were separated by at least 1 coding exon to control for genomic contamination.
(DOCX)

Table S5 **Primers used for detection of cassette exons.** Forward and reverse primers used for detection of novel nesprin-1 and nesprin-2 cassette exons.
(DOCX)

Table S6 **Primers used for the detection of ΔKASH variants.** Forward and reverse primers used for detection of nesprin-1 and nesprin-2 ΔKASH variants.
(DOCX)
