# Supplementary figures and images for: Multiple Novel Nesprin-1 and Nesprin-2 Variants Act as Versatile Tissue-Specific Intracellular Scaffolds
Source: PLoS One. 2012 Jul 2;7(7):e40098. doi: 10.1371/journal.pone.0040098 (PMC3388047; doi:10.1371/journal.pone.0040098)

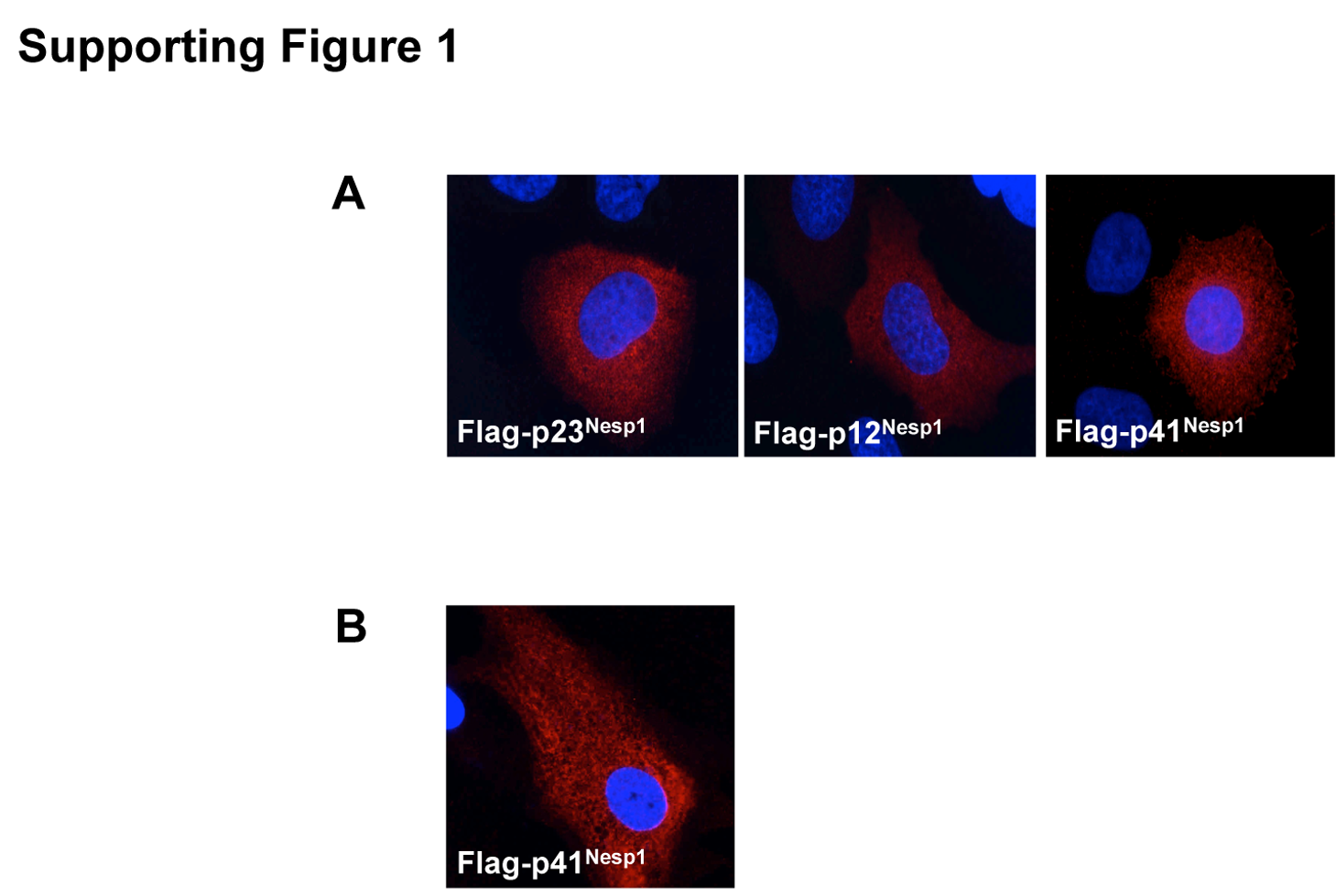

Supplement: Figure S1 — Localizations of p23Nesp1, p12Nesp1 and p41Nesp1. A) p23Nesp1, p12Nesp1 and p41Nesp1 displayed diffusive cytoplasmic localization when transfected into U2OS cells. B) p41Nesp1 displayed diffusive localization and concentrated around the ER when transfected into HDFs. (TIF) [file pone.0040098.s001.tif]

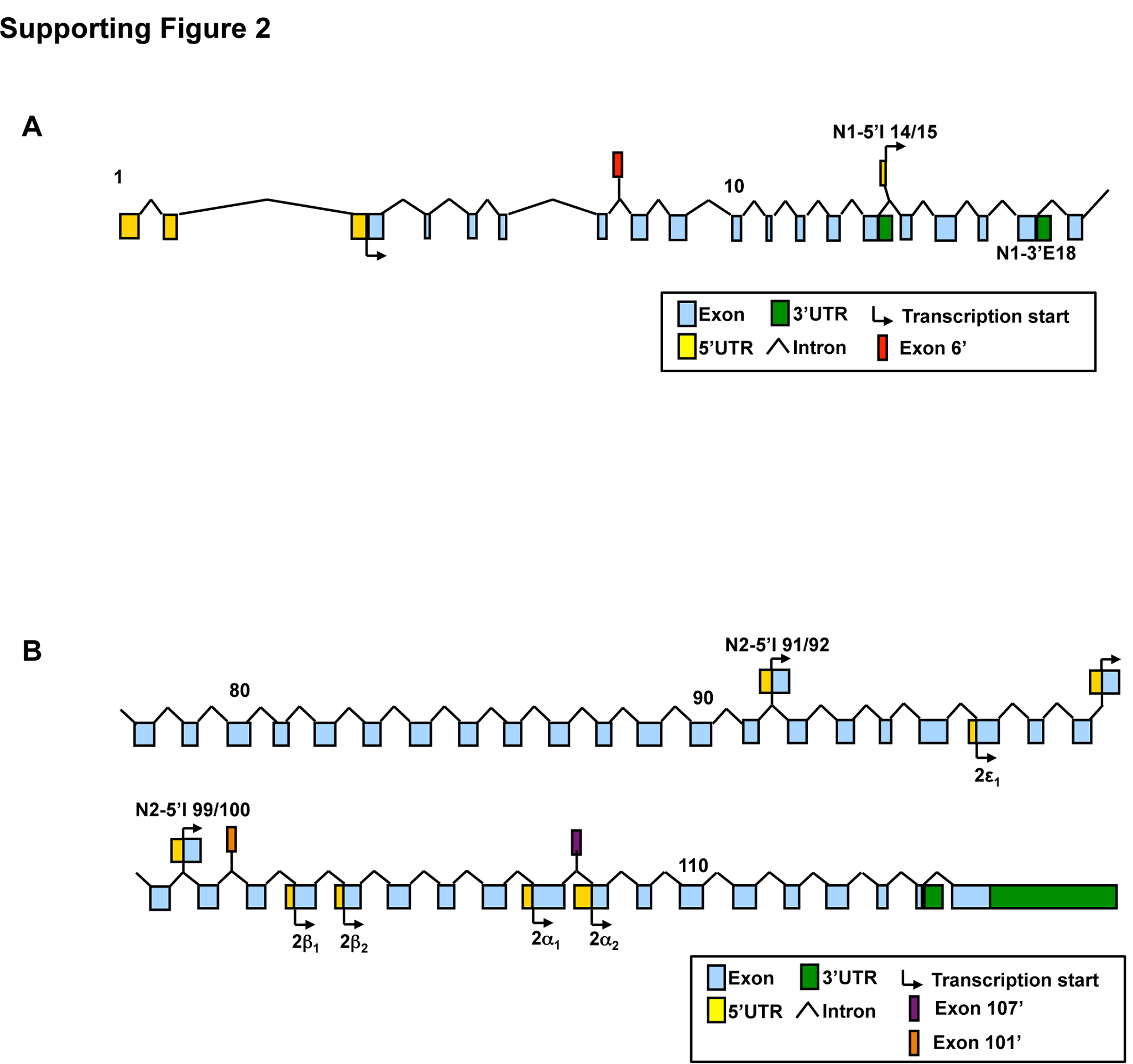

Supplement: Figure S2 — Schematics of nesprin-1 and nesprin-2 cassette exons. A) Nesprin-1 genomic map showing the location of nesprin-1 cassette exon 6′ (Red box). B) Nesprin-2 genomic map showing the location of nesprin-2 cassette exons 101’ (orange box) and107’ (Purple box). (TIF) [file pone.0040098.s002.tif]
