# Supplementary material for: Multiple Novel Nesprin-1 and Nesprin-2 Variants Act as Versatile Tissue-Specific Intracellular Scaffolds
Source: PLoS One. 2012 Jul 2;7(7):e40098. doi: 10.1371/journal.pone.0040098 (PMC3388047; doi:10.1371/journal.pone.0040098)
Supplement: Table S1 — UTR combinations used to generate potential nesprin-1 variants. Nesprin-1 can generate multiple variants through the use of alternative UTRs in a ‘mix-and-match’ approach. The tables highlight the UTR pairs used to generate the potential isoforms described in Figures 2B. (DOCX) [file pone.0040098.s003.docx]

**Table S1**

| **Nesprin-1 Variant** | **5’UTR** | **3’UTR** |
| --- | --- | --- |
| **p603CH^Nesp1^** | Nesprin-1 giant 5’UTR | N1-3’E82 |
| **p391CH^Nesp1^** | Nesprin-1 giant 5’UTR | N1-3’E62 |
| **p252CH^Nesp1^** | Nesprin-1 giant 5’UTR | N1-3’E44 |
| **p81CH^Nesp1^** | Nesprin-1 giant 5’UTR | N1-3’E18 |
| **p56CH^Nesp1^** | Nesprin-1 giant 5’UTR | N1-3’E14 |
| **P55^Nesp1^** | N1-5’I 14/15 | N1-3’E18 |
| **p144^Nesp1^** | N1-5’E83 | N1-3’E106 |
| **p50^Nesp1^** | N1-5’E83 | N1-3’E90 |
| **p12^Nesp1^** | Nesprin-1β_1_ 5’UTR | N1-3’E87 |
| **p53KASH^Nesp1^** | N1-5’E138 | Nesprin-1 giant 3’UTR |
| **p53ΔKASH^Nesp1^** | N1-5’E138 | Nesprin-1 giant 3’UTR |
| **p931KASH^Nesp1^** | N1-5’I 14/15 | Nesprin-1 giant 3’UTR |
| **p346KASH^Nesp1^** | N1-5’E92 | Nesprin-1 giant 3’UTR |
