# Supplementary material for: Multiple Novel Nesprin-1 and Nesprin-2 Variants Act as Versatile Tissue-Specific Intracellular Scaffolds
Source: PLoS One. 2012 Jul 2;7(7):e40098. doi: 10.1371/journal.pone.0040098 (PMC3388047; doi:10.1371/journal.pone.0040098)
Supplement: Table S2 — UTR combinations used to generate potential nesprin-2 variants. Nesprin-2 can generate multiple variants through the use of alternative UTRs in a ‘mix-and-match’ approach. The tables highlight the UTR pairs used to generate the potential isoforms described in Figures 3B. (DOCX) [file pone.0040098.s004.docx]

**Table S2**

| **Nesprin-2 Variant** | **5’UTR** | **3’UTR** |
| --- | --- | --- |
| **p32CH^Nesp2^** | Nesprin-2 giant 5’UTR | N2-3’E9 |
| **p220CH^Nesp2^** | Nesprin-2 giant 5’UTR | N2-3’E46 |
| **p380CH^Nesp2^** | Nesprin-2 giant 5’UTR | N2-3’E50 |
| **p415KASH^Nesp2^** | N2-5’E49 | Nesprin-2 giant 3’UTR |
| **p152KASH^Nesp2^** | N2-5’I 91/92 | Nesprin-2 giant 3’UTR |
