# Supplementary material for: Multiple Novel Nesprin-1 and Nesprin-2 Variants Act as Versatile Tissue-Specific Intracellular Scaffolds
Source: PLoS One. 2012 Jul 2;7(7):e40098. doi: 10.1371/journal.pone.0040098 (PMC3388047; doi:10.1371/journal.pone.0040098)
Supplement: Table S3 — Primers used for 5′ and 3′ RACE. Primers and nested primers used for detection of novel nesprin-1 and nesprin-2 cDNA ends. (DOCX) [file pone.0040098.s005.docx]

**Table S3**

| **UTR** | **Primer** | **Nested Primer** |
| --- | --- | --- |
| **N1-5’E83** | TTCAGCTCTTGCTTCACCAACTTTCCA | TTAGTCTTCACTTTCTCCTGCATGA |
| **N1-3’E44** | AAAACAGACATGGAGAG | ACCGTGGACAAATGGCTGGAT |
| **N1-3’E82** | CTCATGCAGGAAATCACCGCC | ATGCAAGATCGGTGCCTGAA |
| **N1-3’E90** | AGGCCAGCCGGCTGCAGCACAC | CGCCATCCAGCAGTGTAACATCATG |
| **N1-3’E106** | CAAGCTACAGGTCGAGCAGCCGTC | CATGCAAGAACTCTCCAAGCTCCAG |
| **N1-3’E14** | AGGAAACAGCAAACACGATA | CAACGGAAACTTGAGCAACATAAG |
| **N2-3’E50** | GTTGCCACTGTCTTACAGAGAAGC | TTTAGAGCGCTTGGAACAGAGCAAG |
| **N2-3’E90** | TTGGATAGACTTCACCAACAG | GAAAAAGAAAATCCTGACTCA |
