# Supplementary material for: Multiple Novel Nesprin-1 and Nesprin-2 Variants Act as Versatile Tissue-Specific Intracellular Scaffolds
Source: PLoS One. 2012 Jul 2;7(7):e40098. doi: 10.1371/journal.pone.0040098 (PMC3388047; doi:10.1371/journal.pone.0040098)
Supplement: Table S4 — Primers used for UTR detection. Forward and reverse primers used for detection of novel nesprin-1 and nesprin-2 UTRs. Forward and reverse primers were separated by at least 1 coding exon to control for genomic contamination. (DOCX) [file pone.0040098.s006.docx]

**Table S4**

| **UTR** | **Forward Primer** | **Reverse Primer** |
| --- | --- | --- |
| **N1-3’E62** | TGGCTCCCGAAGTGAAACAGAACA | GCGTTGCACTCACAAAGCATCTGA |
| **N1-3’E18** | TCCCAGCCAACCAAGTAAAC | AAATGATGCCCAGGAGACAC |
| **N1-5’I14/15** | AGCACCCAGCTCCTATTCAAGGAT | TCCACTGAGCGGTGGTTTCATTCA |
| **N1-3’E25** | TTGGAGAAGGTACTGCGGATTGCT | AGTTAAATGTTCTTGCACCCTGGA |
| **N1-3’E37** | GCTTCAGAGATACCGCTTGG | CCTTCCTCACACTTCCTTGAAC |
| **N1-5’E138** | CCCACACCTGCCATTTCTAT | CCGTTTGGGTTTCGGTACTA |
| **N1-5’I21/22** | TCAAGCAAGGGTCTCATTCTGGGA | ATCACAGGCACCCTCTGCTCAATA |
| **N1-5’E92** | CCTGCAGGAAGATGAACGCTACTA | ATGGCTCCAATCCCGGTTCATAGA |
| **N1-5’I128/129** | AGTCCTGAATGGCAACATCC | GCAAAGTTGTTGGCCTGAGT |
| **N1-5’E83** | GGGTTCCTTTCACTTCACTTCTGT | ACTTCAGCCAACTGAAGGGAGAGT |
| **N1-3’E44** | TGCCAGCAGTGTGATTGTAACCAG | AGCCTGGGCAACAAGAGTGAAAC |
| **N1-3’E82** | TGGATGAGTGGACGGGCTTTAACA | TGAGCCGAGATCACACATTTGCAC |
| **N1-3’E90** | AGTTGGACGTCTCAGTCTCAAGGA | TTTGATGGCTGAGCCCACACAATG |
| **N1-3’E106** | TGGGCAGGTTATCCTTGCTAGACT | CTGCAGCTGAATACTACTGGCTGA |
| **N1-3’E14** | ACAATCTTGGGATAGAGTGACCTCC | AGTAGGGCTGTTATGCTGCAAGGT |
| **N2-3’E50** | AGCATTTCAGGAGCAAGTTTGGGC | GCCTCTGCCAGTTTGTTCTGCAAT |
| **N2-3’E90** | TCTCCAGAGGGCTGCTTATTTGGA | ACAGAGCGAGAGTCCGTCTCAAA |
| **N2-3’E9** | CGCCACCTATGAGTCTGTCA | GCAGAACTTCTCCAGGCATC |
| **N2-3’E46** | GCTAAGCAGGAGATGGAATGTTGTC | CCAAATTGATCATCAGGCAAAGGT |
| **N2-5’E49** | ACTCAGAGACATATTATCTATGTACCACG | CTTGCTCTGTTCCAAGCGCTCTAA |
| **N2-5’I 91/92** | AATTCCACCTACTTGGGAGGCTGA | TGGGAGCTTGAGGCTCACTTCATT |
| **N2-5’I 99/100** | GGAACTTGGAGCTTTCGCTTA | GTGCAGTAGGACGTCACAGATGTT |
