# Supplementary material for: Multiple Novel Nesprin-1 and Nesprin-2 Variants Act as Versatile Tissue-Specific Intracellular Scaffolds
Source: PLoS One. 2012 Jul 2;7(7):e40098. doi: 10.1371/journal.pone.0040098 (PMC3388047; doi:10.1371/journal.pone.0040098)
Supplement: Table S5 — Primers used for detection of cassette exons. Forward and reverse primers used for detection of novel nesprin-1 and nesprin-2 cassette exons. (DOCX) [file pone.0040098.s007.docx]

**Table S5**

| **Splicing** | **Forward Primer** | **Reverse Primer** |
| --- | --- | --- |
| **Nesprin-1 Exon 6’** | TAAACTGCTTGCCCTTCTGGAGGT | ATCCAAGAACTATTGAGGGTCGGC |
| **Nesprin-1 Exon 93** | ATGCTGACGATGAAAGCCAAGCAC | TTCCCAAGAACGCTGCAAATCACC |
| **Nesprin-2 Exon 28-31** | AGGACAGGAGTAGTTCTTGTCTGC | TCTCCTTTCCCATGTACGAAGCCT |
| **Nesprin-2 Exon 101’** | TGAGTCTGAGCTTTCCAAGCCTGT | AGCTGACTTGAGCCAGTCCTCAAA |
| **Nesprin-2 Exon 107’** | ATCTCCTCAGTCCCTGTGTCATCT | TTGCTTGTAGTGATGCTCGGGACA |
| **Nesprin-2 Exon 110-113** | AAGCTACTATTACCTCCAGGCACG | AGTCTACCTCGTCGAAGCTGGGCA |
| **Nesprin-2 Exon 114** | TACGGGAGCAAGTGTCCCAAGATT | GCCTTCTGTAGTTCTCACTGC |
