# Supplementary material for: Multiple Novel Nesprin-1 and Nesprin-2 Variants Act as Versatile Tissue-Specific Intracellular Scaffolds
Source: PLoS One. 2012 Jul 2;7(7):e40098. doi: 10.1371/journal.pone.0040098 (PMC3388047; doi:10.1371/journal.pone.0040098)
Supplement: Table S6 — Primers used for the detection of ΔKASH variants. Forward and reverse primers used for detection of nesprin-1 and nesprin-2 ΔKASH variants. (DOCX) [file pone.0040098.s008.docx]

**Table S6**

| **ΔKASH** | **Forward Primer** | **Reverse Primer** |
| --- | --- | --- |
| **Nesprin-1ΔKASH** | GACAGAAAACGGTCCACAAAAGG | TCGCCAAGATCAAGGTCCTCTTGT |
| **Nesprin-2ΔKASH1** | AAGCTACTATTACCTCCAGGCACG | AGTCTACCTCGTCGAAGCTGGGCA |
| **Nesprin-2 ΔKASH2** | CCAGCTTCGACGAGGTAGAC | CAGCCCTTTCCAGACAAAAG |
